# Co-enrichment of CD8-positive T cells and macrophages is associated with clinical benefit of tislelizumab in solid tumors

**DOI:** 10.1186/s40364-023-00465-w

**Published:** 2023-03-07

**Authors:** Dingwei Ye, Jayesh Desai, Jingwen Shi, Si-Yang Maggie Liu, Wei Shen, Tengfei Liu, Yang Shi, Dan Wang, Liang Liang, Silu Yang, Xiaopeng Ma, Wei Jin, Pei Zhang, Ruiqi Huang, Zhirong Shen, Yun Zhang, Yi-Long Wu

**Affiliations:** 1 Department of Urology, Fudan University Shanghai Cancer Center, Shanghai, China; 2 Department of Medical Oncology, Peter MacCallum Cancer Centre and the University of Melbourne, Melbourne, Australia; 3 Clinical Biomarkers, BeiGene (Beijing) Co., Ltd., 6 Jianguomenwai Avenue, Central International Trade Center, 18th Floor, Tower D Chaoyang District, Beijing, 100022 China; 4 Department of Hematology, First Affiliated Hospital, The Clinical Medicine Postdoctoral Research Station, Jinan University, Guangzhou, China; 5 Department of Statistics, BeiGene (Shanghai) Co., Ltd., Shanghai, China; 6 Department of Pulmonary Oncology, Guangdong Lung Cancer Institute, Guangdong Provincial People’s Hospital, Guangdong Academy of Medical Sciences, Guangzhou, 51008 China

**Keywords:** Immunotherapy, Tumor microenvironment, Macrophages, Immune cells, Tislelizumab

## Abstract

**Background:**

Activated immune cells (IC) in the tumor microenvironment (TME) are critical for anti-tumor efficacy. Greater understanding of the dynamic diversity and crosstalk between IC is needed to clarify their association with immune checkpoint inhibitor efficacy.

**Methods:**

Patients from three tislelizumab monotherapy trials in solid tumors (NCT02407990, NCT04068519, NCT04004221) were retrospectively divided into subgroups by CD8^+^ T-cell and macrophage (Mφ) levels, assessed via multiplex immunohistochemistry (mIHC; *n* = 67) or gene expression profiling (GEP; *n* = 629).

**Results:**

A trend of longer survival was observed in patients with both high CD8^+^ T-cell and Mφ levels versus other subgroups in the mIHC analysis (*P* = 0.11), which was confirmed with greater statistical significance in the GEP analysis (*P* = 0.0001). Co-existence of CD8^+^ T cells and Mφ was coupled with elevated CD8^+^ T-cell cytotoxicity, T-cell trafficking, MHC class I antigen presentation signatures/genes, and enrichment of the pro-inflammatory Mφ polarization pathway. Additionally, a high level of pro-inflammatory CD64^+^ Mφ density was associated with an immune-activated TME and survival benefit with tislelizumab (15.2 vs. 5.9 months for low density; *P* = 0.042). Spatial proximity analysis revealed that closer proximity between CD8^+^ T cells and CD64^+^ Mφ was associated with a survival benefit with tislelizumab (15.2 vs. 5.3 months for low proximity; *P* = 0.024).

**Conclusions:**

These findings support the potential role of crosstalk between pro-inflammatory Mφ and cytotoxic T cells in the clinical benefit of tislelizumab.

**Trial registration:**

NCT02407990, NCT04068519, NCT04004221.

**Supplementary Information:**

The online version contains supplementary material available at 10.1186/s40364-023-00465-w.

## Introduction

In recent years, immunotherapy has transformed how we approach the treatment of multiple cancers. In particular, therapies targeting the programmed cell death protein 1 (PD-1) receptor and programmed death-ligand 1 (PD-L1) pathway are providing treatment opportunities in a wide range of tumor types by modulating the immune system to control tumor growth [1, 2]. However, not all patients benefit from PD-(L)1 blockade, and the most widely used biomarker, PD-L1, remains limited in its predictive capacity, highlighting an urgent need for novel biomarkers to identify patients who will benefit from these agents. CD8-positive (CD8^+^) T cells infiltrating the tumor microenvironment (TME) are considered the key tumor killing cells and main target of the PD-1/L1 axis and have thus been proposed to be predictive of the clinical efficacy of immunotherapy [3, 4]. However, given the heterogeneity and complexity of the TME, exploration of the function of other cell types in the TME and their interplay with CD8^+^ T cells is critical for a deeper mechanistic understanding and to guide future clinical use. Thus far, the presence of large numbers of fibroblasts, myeloid-derived suppressor cells, and macrophages (Mφ) has been demonstrated in the TME, all of which exert either positive or negative regulation on CD8^+^ T cells [5].

Innate immunity, which is dominated by myeloid cells including Mφ, plays an important role in the TME [6]. Upon the onset of tumor niche development, Mφ are typically recruited from the circulation and are subsequently directed by various signals (e.g., cytokines) from tumor and stromal cells in the TME [7, 8]. According to the classical concept, Mφ can be dichotomized into M1-type and M2-type activation states associated with pro-inflammatory and anti-inflammatory functions, respectively [9, 10]. Because of their functional versatility, the prognostic role of Mφ remains controversial. M2-type Mφ impair CD8^+^ T-cell cytotoxic function by expressing immune checkpoint ligands and produce immune-suppressive cytokines [11]. Mφ can recruit T cells into the TME by secreting pro-inflammatory chemokines and directly releasing tumor-killing molecules such as reactive oxygen species and nitric oxide [12, 13].

However, a more contemporary understanding of Mφ types suggests no absolute boundary between M1- and M2-type Mφ, and flexibility between different states has been observed [9, 14]. Mφ exhibit a spectrum of phenotypes, not only with respect to biological function, but also in terms of gene expression profiles and cell surface markers [9]. The complexity of Mφ properties is further supported by the recent rise of single-cell RNA-sequencing (RNA-seq) technology, which has been used to identify multiple clusters/populations of Mφ with distinct gene expression profiles in solid tumors [15, 16]. Among these clusters, C1QC^+^ Mφ have been shown to exhibit significantly higher phagocytosis signatures and ISG15^+^ Mφ were associated with higher canonic pro-inflammatory M1 signatures, while SPP1^+^ pro-angiogenic Mφ functioned conversely and were typically associated with poor prognosis; however, there are limited data for Mφ with strong proliferative or regulatory features in the TME [17].

Mφ may influence anti-tumor responses further through the expression of multiple Fc gamma receptors (FcγRs), which may interfere with drug treatment through therapeutic antibody binding and subsequent antibody-dependent cellular phagocytosis (ADCP). In particular, FcγRI (CD64) is reported to be induced by interferon-gamma (IFNγ) signaling, and therefore is highly expressed on IFNγ-induced M1-type, pro-inflammatory Mφ [18–21]. CD64-positive (CD64^+^) Mφ exhibit an increased phagocytosis ability [22], which has been reported to be associated with blocking the anti-tumor effect of anti-PD-1 antibodies in mouse models through ADCP-mediated T-cell elimination [23]. The anti-PD1 antibody tislelizumab has been specifically designed with mutations in the Fc region to minimize binding to CD64 on Mφ [23]. We therefore investigated the association between CD64^+^ Mφ and clinical outcomes in patients treated with tislelizumab.

In order to comprehensively dissect the roles and phenotypes of CD8^+^ T cells and Mφ in the TME, we applied multiplex immunohistochemistry (mIHC) and gene expression profiling (GEP) using both CD8^+^ T-cell and Mφ markers, including CD64 and CD68, to baseline samples collected from three clinical trials of tislelizumab monotherapy. Associations with clinical outcomes, potential molecular mechanisms, and crosstalk between immune cells are reported herein.

## Materials and methods

### Clinical cohorts and data collection

Patient data were collected from three clinical studies of tislelizumab monotherapy: A317-001 (NCT02407990) [24], A317-102 (NCT04068519) [11], and A317-204 (NCT04004221) [25]. A317-001 and A317-102 were Phase 1/2 studies in multiple cancer types conducted globally and in China, respectively. A317-204 was a Phase 2 study conducted in China and Korea in patients with previously treated, PD-L1‒positive (PD-L1 +) urothelial carcinoma. Ethical approval was obtained from the relevant institutional review boards and all procedures followed were in accordance with the ethical standards of the responsible committee on human experimentation (institutional and national) and with the Helsinki Declaration of 1964 and later versions. Informed consent to be included in the study, or the equivalent, was obtained from all patients. Baseline formalin-fixed, paraffin-embedded (FFPE) samples were collected for biomarker testing. Overall survival (OS) of tislelizumab-treated patients in the biomarker-evaluable population (BEP) from the three studies was pooled and analyzed to explore the association with biomarker subgroups in this retrospective analysis.

### mIHC and data analysis

mIHC was performed using an Opal automation mIHC kit (PerkinElmer NEL801001KT or NEL821001KT, or equivalent) on the Leica BOND Rx platform followed by IF 6-colorWJJ-CD30 protocol in a CAP-controlled area within the Oncology and Immunology Unit of WuXi AppTec. Human FFPE specimens were labeled with different primary antibodies (CD64/FcγRΙ OTI3D3, Abcam ab140779; CD68 KP-1, VENTANA 790–2931; PD-L1 SP263, VENTANA 790–4905; CD8 SP57, VENTANA 790–4460; pan-Keratin AE1/AE3/PCK26, VENTANA 760–2135), followed by appropriate secondary antibodies (Polymer HRP from Opal automation mIHC kit) and different Opal dyes, and finally counterstained with spectral 4’,6-diamidino-2-phenylindole (DAPI). Rabbit immunoglobulin G (IgG) (Abcam ab172730, EPR25A) and mouse immunoglobulin IgG1 (Abcam ab18443, kappa monoclonal MOPC-21) were used as the isotype control. Whole-slide images were acquired for each patient using the Leica Aperio VERSA 8 automated microscope. Image analysis was performed using the HALO software package (Indica Labs, Albuquerque, NM, USA), and the segmentation and mark-up of individual cells were performed, reviewed, and scored by two pathologists in a blinded manner using the HALO HighPlex FL module (Indica Labs). “High” and “low” immune cell density subgroups were defined using the median density as the cutoff between groups.

To determine the spatial relationship between CD8^+^ T cells and CD64^+^ Mφ, the Spatial Analysis Module of HALO was used. The proximity algorithm works by calculating the number of cells within a given distance of another cell. The average number of CD64^+^CD68^+^ cells ≤ 30 μm from each CD8^+^ T cell was determined across the total tumor area.

### Gene expression profiling

Gene expression data were generated using the HTG EdgeSeq Precision Immuno-Oncology Panel (HTG Molecular Diagnostics, Inc., Tucson, AZ, USA), per the manufacturer’s instructions. The library was sequenced on the Illumina Nextseq 500 platform (Illumina, Inc., San Diego, CA, USA) and data were processed by HTG EdgeSeq parser software. Read count was normalized by library size to obtain count per million, which was then log transformed for downstream analysis [26]. “High” and “low” gene expression subgroups were defined using the median expression as the cutoff between groups.

### The Cancer Genome Atlas (TCGA) data analysis

Gene expression and clinical data of 8485 solid tumors were retrieved from the Genomic Data Commons data portal (https://portal.gdc.cancer.gov/). The value of the fragments per kilobase of transcript per million mapped reads after upper-quartile normalization was used for signature score and survival analyses. “High” and “low” gene expression subgroups were defined using the median expression as the cutoff between groups.

### Differential gene expression and gene set enrichment analysis (GSEA)

Differentially expressed genes or gene signatures were identified using the limma-voom workflow [27]. A normalized gene signature score for each individual sample was calculated using gene set variation analysis (GSVA) package [26]. In the GEP dataset analysis, gene sets from Jerby-Arnon and colleagues [28] and Tirosh and colleagues [29] were used to estimate the abundance of CD8^+^ T cells and Mφ, respectively; 29 gene signature sets were used to describe the immune status and tumor features in the TME [30]. In addition, for gene set enrichment analysis (GSEA), a gene list ranked according to the log fold change was used [31].

### Single-cell RNA-seq datasets

The expression level of Mφ-related genes was retrieved from the single-cell RNA-seq Data Visualization and Analyzation tool (Peking University, Beijing, China) (http://cancer-pku.cn:3838/Pan_Myeloid/). The Mφ subtype definition from Cheng and colleagues was used [17]. A cell was defined as positive for a gene if it was covered by at least one read, and the percentage of positive cells in each subtype was defined as the “proportion”. The normalized value from the initial publication was then used to calculate the average expression of each gene across subtypes.

### Statistical analysis

Median OS was estimated by the Kaplan–Meier method and a log-rank test was used to compare survival curves between different biomarker-defined patient subgroups throughout the study if there was no other specification. In the GEP dataset analysis only, a Cox model was applied to investigate the association of the composed biomarkers (CD8^Hi^/Mφ^Hi^ vs. others) with OS, and the impact of cancer type was also evaluated by adjusting it in the model. Hazard ratio (HR) and 95% confidence intervals (CI) were estimated from the models. To compare median gene expression or signature scores between biomarker-defined subgroups, Wilcoxon rank‒sum test was used. All statistical analyses and visualizations were performed in R (v.4.0). All *P*-values are descriptive as this is a post-hoc exploratory analysis.

## Results

### High density of CD8^+^ T cells and Mφ is associated with a trend towards longer OS in patients receiving tislelizumab treatment

**Fig. 1:**
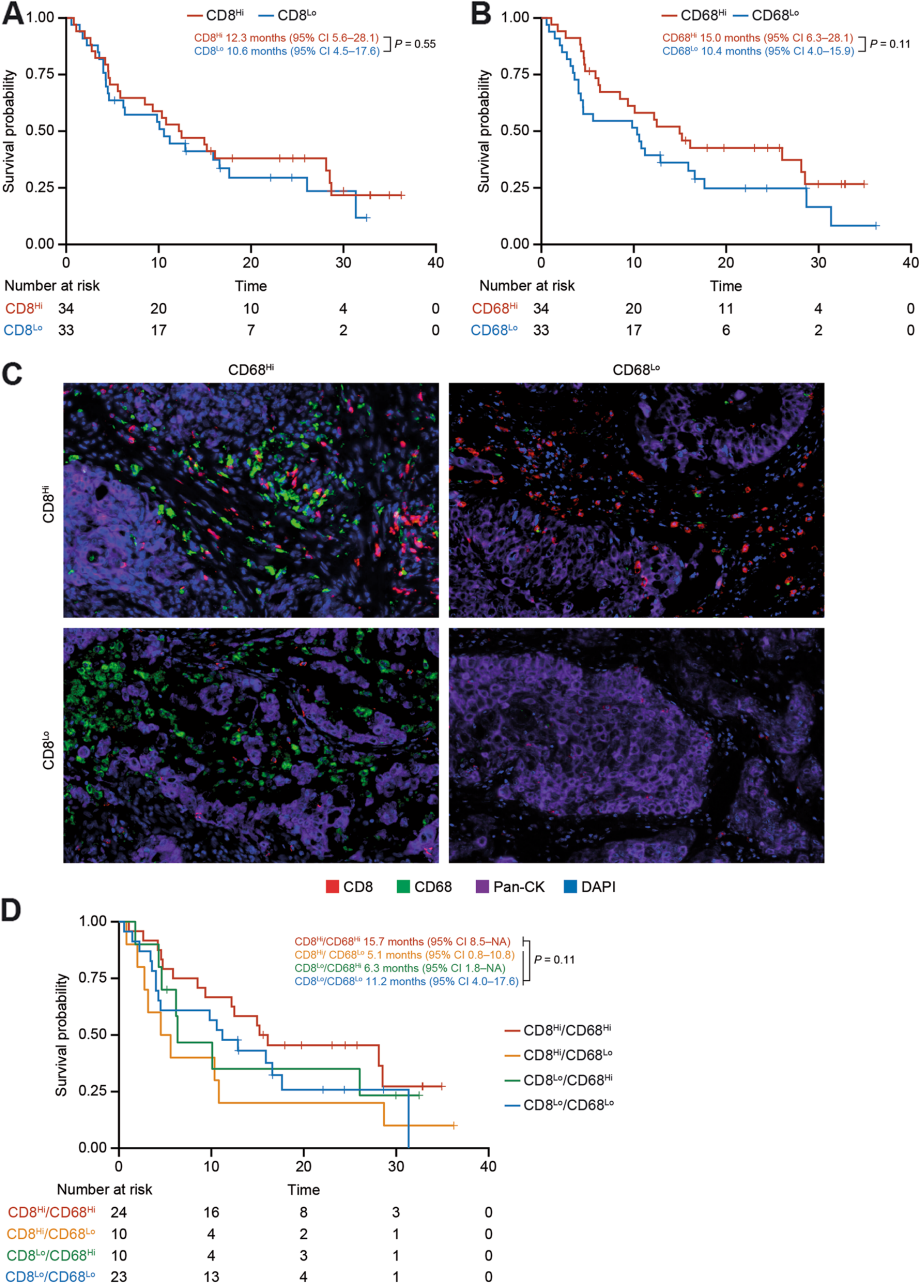
Association of mIHC-defined immune cells with survival benefit of tislelizumab treatment **A**–**B** Kaplan–Meier overall survival analysis in subgroups defined by density of CD8^+^ T cells (**A**) and CD68^+^ Mφ (**B**) in mIHC BEP. **C** Representative mIHC image for four subgroups defined by density of CD8^+^ T cells and CD68^+^ Mφ. Scale bar: 50 µm. **D** Kaplan–Meier overall survival analysis in four subgroups defined by density of CD8^+^ T cells and CD68^+^ Mφ. Median overall survival was estimated by the Kaplan–Meier method and the log-rank test was used to compare survival curves between defined biomarker subgroups. BEP, biomarker-evaluable population; CI, confidence interval; DAPI, 4’,6-diamidino-2-phenylindole; Mφ, macrophages; mIHC, multiplex immunohistochemistry; NA, not available; Pan-CK, pan-cytokeratin

To assess the association of CD8^+^ T cells, Mφ, and PD-L1 expression and response to tislelizumab, an mIHC panel was developed including CD8 (cytotoxic T cells), CD68 (tumor-associated Mφ), CD64, PD-L1, and Pan-cytokeratin (tumor cell) surface markers to identify cell subtypes in the TME (Supplemental Fig. 1A). To explore the association between cell components and OS in patients treated with tislelizumab, patients (*n* = 67) were assigned to subgroups according to the density of PD-L1 + cells, PD-L1 + tumor cells, PD-L1 + Mφ, CD8^+^ T cells, and Mφ. Patients with a high density of PD-L1 + cells, PD-L1 + tumor cells, or PD-L1 + Mφ exhibited no significant OS difference from those with a low density (Supplemental Fig. 1B–D). Comparable median OS was observed in patients with high CD8^+^ T-cell density (CD8^Hi^) compared with patients with low CD8^+^ T-cell density (CD8^Lo^) (12.3 months vs. 10.6 months, *P* = 0.55; Fig. 1A). More prominently, patients with high Mφ density (CD68^Hi^) showed a trend towards a longer median OS compared with patients with low Mφ density (CD68^Lo^), although this did not reach statistical significance (15.0 months vs. 10.4 months, *P* = 0.11; Fig. 1B).

Given the different functions and potential crosstalk between CD8^+^ T cells and Mφ in the TME, we explored the clinical benefit of tislelizumab in patients categorized into four subgroups according to the density of CD8^+^ T cells and Mφ (Fig. 1C), using a median cut-off. Although the analysis was limited by small sample size and insufficient statistical power, patients with CD8^Hi^/CD68^Hi^ showed the longest median OS (15.7 months) compared with the other three subgroups (5.1, 6.3, and 11.2 months for CD8^Hi^/CD68^Lo^, CD8^Lo^/CD68^Hi^, and CD8^Lo^/CD68^Lo^, respectively; CD8^Hi^/CD68^Hi^ vs. others, *P* = 0.11; Fig. 1D).

### CD8^Hi^/Mφ^Hi^ gene signatures are associated with longer OS in patients receiving tislelizumab treatment

**Fig. 2:**
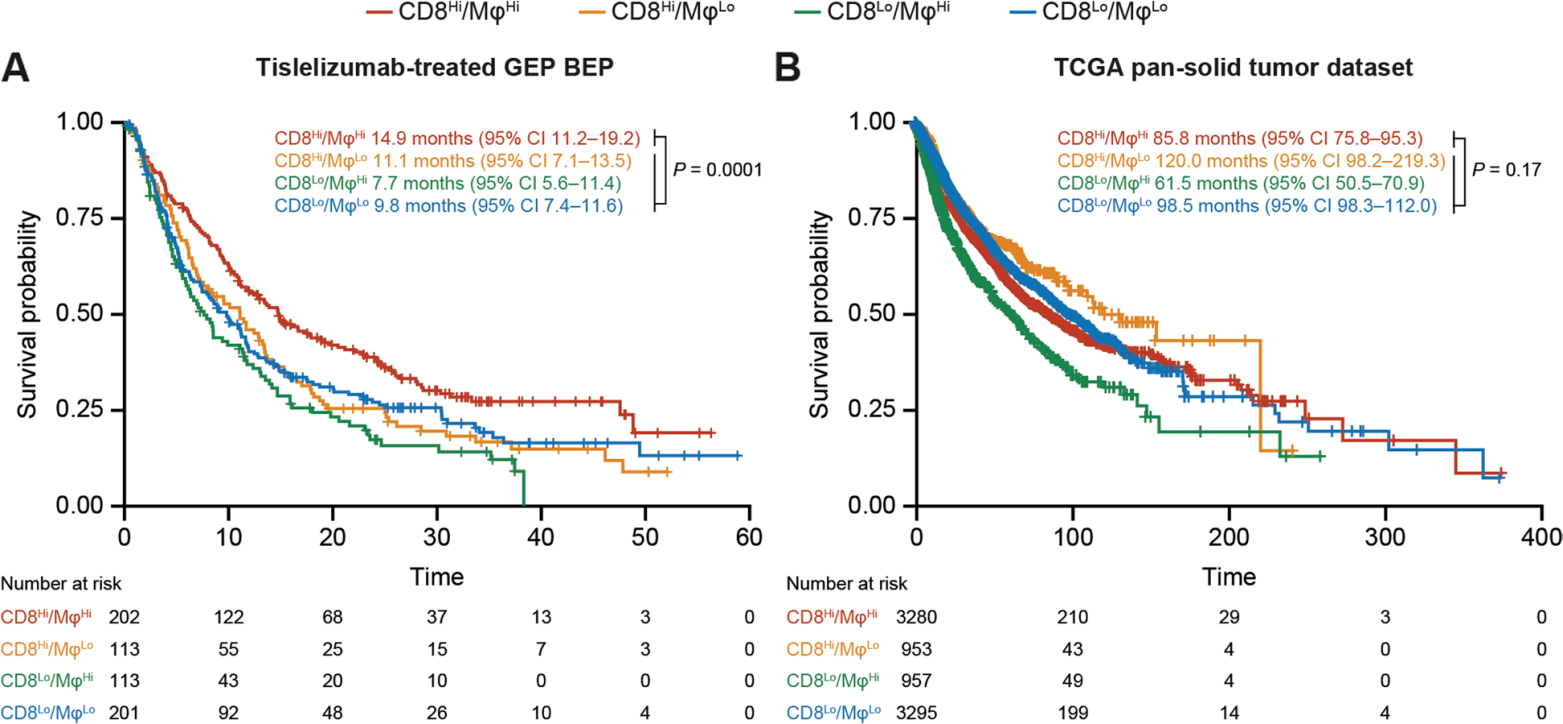
Overall survival in CD8^+^ T-cell and Mφ gene signature-defined subgroups. **A**–**B** Kaplan–Meier overall survival analysis in four subgroups defined by CD8^+^ T-cell signature and Mφ signature in (**A**) tislelizumab-treated GEP BEP and (**B**) TCGA pan-solid tumor dataset. Median overall survival was estimated by the Kaplan–Meier method and the log-rank test was used to compare survival curves between defined biomarker subgroups. BEP, biomarker-evaluable population; CI, confidence interval; GEP, gene expression profiling; Mφ, macrophages; TCGA, The Cancer Genome Atlas

To further explore the observations made in the mIHC BEP (*n* = 67), an additional analysis of a larger population of tislelizumab-treated patients with evaluable GEP (*n* = 629) was conducted. The populations overlapped, with 65 patients from the mIHC BEP included in the GEP BEP. The baseline characteristics and median OS in the GEP BEP and mIHC BEP are shown in Table 1, alongside those from the overall pooled study population. Baseline characteristics and median OS were comparable; however, compared with the mIHC BEP, the GEP BEP included a broader spectrum of different cancer types. Using the median gene signature score of CD8^+^ T cells [28] and Mφ [29] as a cutoff, patients were stratified into four biomarker subgroups. Consistent with the data observed in the mIHC cohort, median OS was longer (14.9 months) in patients with CD8^Hi^/Mφ^Hi^ compared with the other three subgroups (11.1, 7.7, and 9.8 months for CD8^Hi^/Mφ^Lo^, CD8^Lo^/Mφ^Hi^, and CD8^Lo^/Mφ^Lo^, respectively; CD8^Hi^/Mφ^Hi^ vs. others, *P* = 0.0001; Fig. 2A). Additionally, the Cox model also indicated that subgroups with high expression of both biomarkers had an OS advantage compared to others (CD8^Hi^/Mφ^Hi^ vs. others, HR = 0.68 (95% CI 0.55–0.83), unadjusted *P* < 0.0001). In particular, the superiority was consistently observed when adjusting cancer type as a covariate in the Cox model (CD8^Hi^/Mφ^Hi^ vs. others, HR = 0.71 (95% CI 0.57–0.87), adjusted *P* < 0.0001). However, in the TCGA pan-solid tumor dataset or individual dataset of the major indications included in this study (data not shown), patients with CD8^Hi^/Mφ^Hi^ did not exhibit prolonged OS compared with the other subgroups (CD8^Hi^/Mφ^Hi^ vs. others, *P* = 0.17; Fig. 2B), which indicated that the OS benefit observed may potentially be related to tislelizumab rather than a prognostic factor.

**Table 1:** Baseline characteristics and overall survival for the overall population, GEP BEP, and mIHC BEP

Characteristic	Overall^a^ (*N* = 864)	GEP BEP (*n* = 629)	mIHC BEP (*n* = 67)
Median (range) age, years	60 (18–82)	60 (19–81)	59 (26–78)
Sex, n (%)			
Male	537 (62.2)	396 (63.0)	45 (67.2)
Female	327 (37.8)	233 (37.0)	22 (32.8)
ECOG performance status, n (%)			
0	302 (35.0)	228 (36.2)	20 (29.9)
1	562 (65.0)	401 (63.8)	47 (70.1)
Cancer type, n (%)			
NSCLC	105 (12.2)	57 (9.1)	25 (37.3)
GC	78 (9.0)	58 (9.2)	13 (19.4)
EC	79 (9.1)	66 (10.5)	4 (6.0)
UC	152 (17.6)	127 (20.2)	25 (37.3)
HCC	68 (7.9)	50 (7.9)	0 (0.0)
Other	382 (44.2)	271 (43.1)	0 (0.0)
Patients with any prior anticancer drug therapy, n (%)			
0–1	407 (47.1)	297 (47.2)	29 (43.3)
2	204 (23.6)	147 (23.4)	20 (29.9)
≥ 3	190 (22.0)	137 (21.8)	18 (26.9)
Unknown	63 (7.3)	48 (7.6)	0 (0.0)
Median overall survival, months (95% CI)	11.1 (9.5–11.7)	11.1 (9.6–11.9)	11.2 (6.2–16.1)

*BEP* biomarker-evaluable population, *CI* confidence interval, *EC* esophageal cancer, *ECOG* Eastern Cooperative Oncology Group, *GC* gastric cancer, *GEP* gene expression profiling, *HCC* hepatocellular carcinoma, *mIHC* multiplex immunohistochemistry, *NSCLC* non-small cell lung cancer, *UC* urothelial cancer

^a^All patients enrolled in A317-001 (NCT02407990), A317-102 (NCT04068519), and A317-204 (NCT04004221)

### Co-enrichment of CD8^+^ T cells and Mφ contribute to an immune-activated TME

**Fig. 3:**
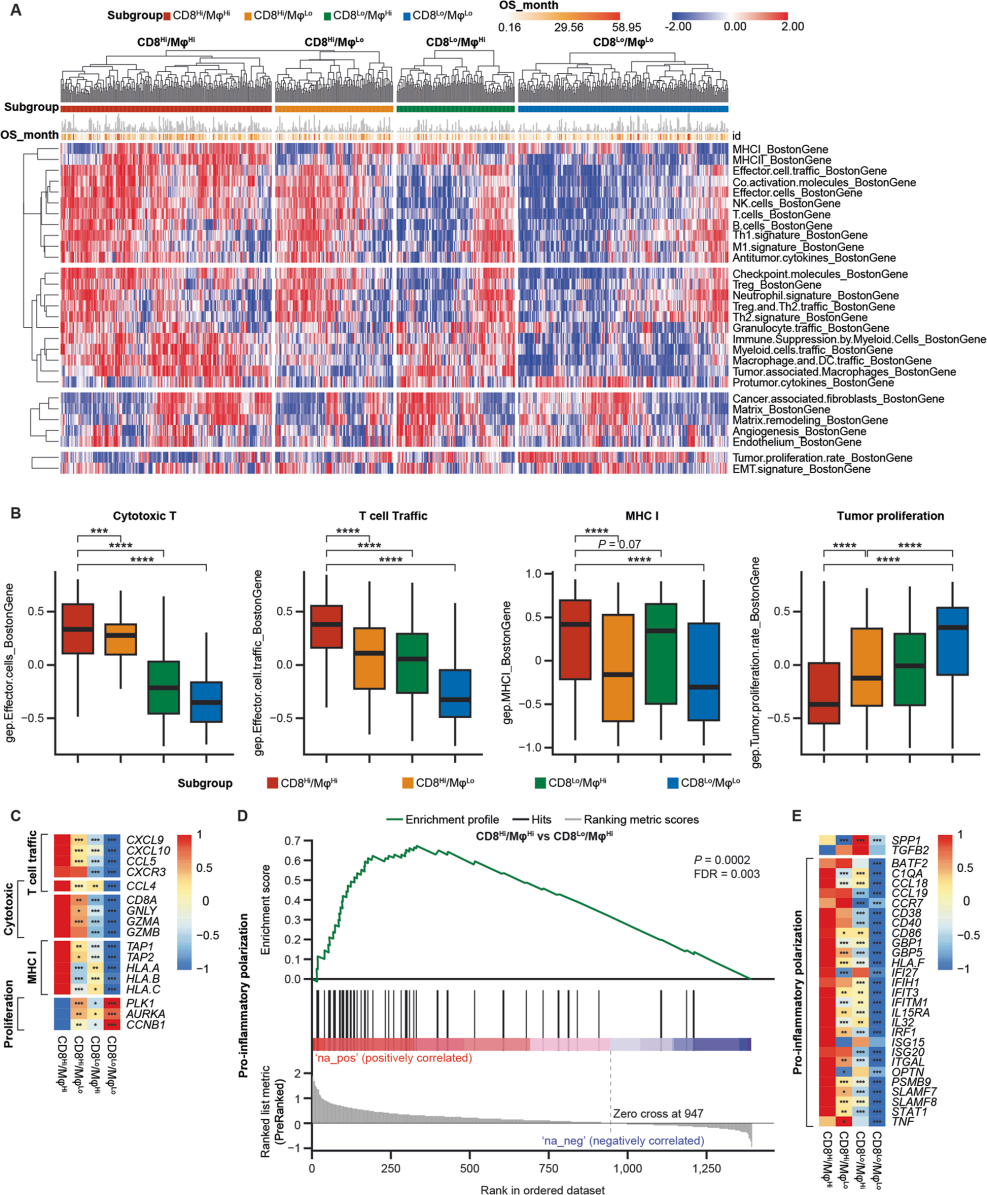
Distinct TME in four signature-defined subgroups in the GEP BEP. **A** Heatmap of 29 key TME-described gene signatures. **B** Box plot showing signature score differences in cytotoxic T-cell, T-cell traffic, MHC I, and tumor proliferation signatures among four subgroups defined by CD8^+^ T-cell and Mφ signatures. **C** Heatmap showing the differentially expressed genes among four subgroups defined by CD8^+^ T-cell and Mφ signatures. **D** GSEA of pro-inflammatory Mφ polarization signal between CD8^Hi^/Mφ^Hi^ and CD8^Lo^/Mφ^Hi^ subgroups. E: Heatmap showing the differentially expressed genes associated with pro-inflammatory Mφ polarization among four subgroups defined by CD8^+^ T-cell and Mφ signatures. *P < .05, **P < .01, ***P < .001, ****P < .0001. BEP, biomarker-evaluable population; FDR, false discovery rate; GSEA, gene set enrichment analysis; Mφ, macrophages; MHC I, major histocompatibility complex class I; OS, overall survival; TME, tumor microenvironment

Differences in the characteristics of the TME in the subgroups defined by levels of CD8^+^ T-cell and CD68^+^ Mφ signatures were subsequently explored. The TME was characterized using 29 functional gene signatures, which included signatures for immune cells, angiogenesis, fibrosis, and malignant cell properties [30]. Subgroups with CD8^Hi^ signatures, regardless of Mφ expression, tended to have an immune-activated TME, with enriched T-cell, cytotoxic cell, B-cell, natural killer cell, regulatory T-cell, and neutrophil signatures compared with CD8^Lo^ subgroups (Fig. 3A). In particular, patients in the subgroup with CD8^Hi^/Mφ^Hi^ signatures had further increased expression of immune-related signatures and genes, including those related to T-cell cytotoxicity (*CD8A, GNLY, GZMA, GZMB*), T-cell trafficking (*CXCL9, CXCL10, CCL4, CCL5*), and major histocompatibility complex class I (MHC I) (*TAP1, TAP2, HLA.A, HLA.B, HLA.C*); in contrast, these patients exhibited the lowest expression of tumor proliferation signatures (*PLK1, AURKA, CCNB1*) (Fig. 3B, C). These analyses demonstrated a more immune-activated TME in this Mφ co-enriched CD8^Hi^ subgroup than in the subgroups without Mφ co-enrichment or with CD8^Lo^ signatures. To further examine Mφ phenotypes with or without CD8^+^ T-cell co-enrichment, we performed GSEA between the CD8^Hi^/Mφ^Hi^ and CD8^Lo^/Mφ^Hi^ subgroups. A significantly higher level of pro-inflammatory polarization signals [16, 32] (e.g. *STAT1, SLAMF7/8, ISG15, IRF1, IL32, CCL18*) and lower expression of pro-angiogenic genes [17] (*SPP1, TGFB2*) was observed in patients with CD8^Hi^/Mφ^Hi^ (*P* = 0.0002) compared with patients with CD8^Lo^/Mφ^Hi^ (Fig. 3D, E).

Taken together, patients with CD8^Hi^/Mφ^Hi^ exhibited the highest anti-tumor phenotype among subgroups defined by cytotoxic T cells and Mφs, which may contribute to an OS benefit following treatment with tislelizumab. To further confirm this observation, we assessed the gene expression signatures in mIHC-defined subgroups and similar findings were observed (Supplemental Fig. 2A, B).

### CD64^+^ Mφ are associated with clinical benefit of tislelizumab treatment

**Fig. 4:**
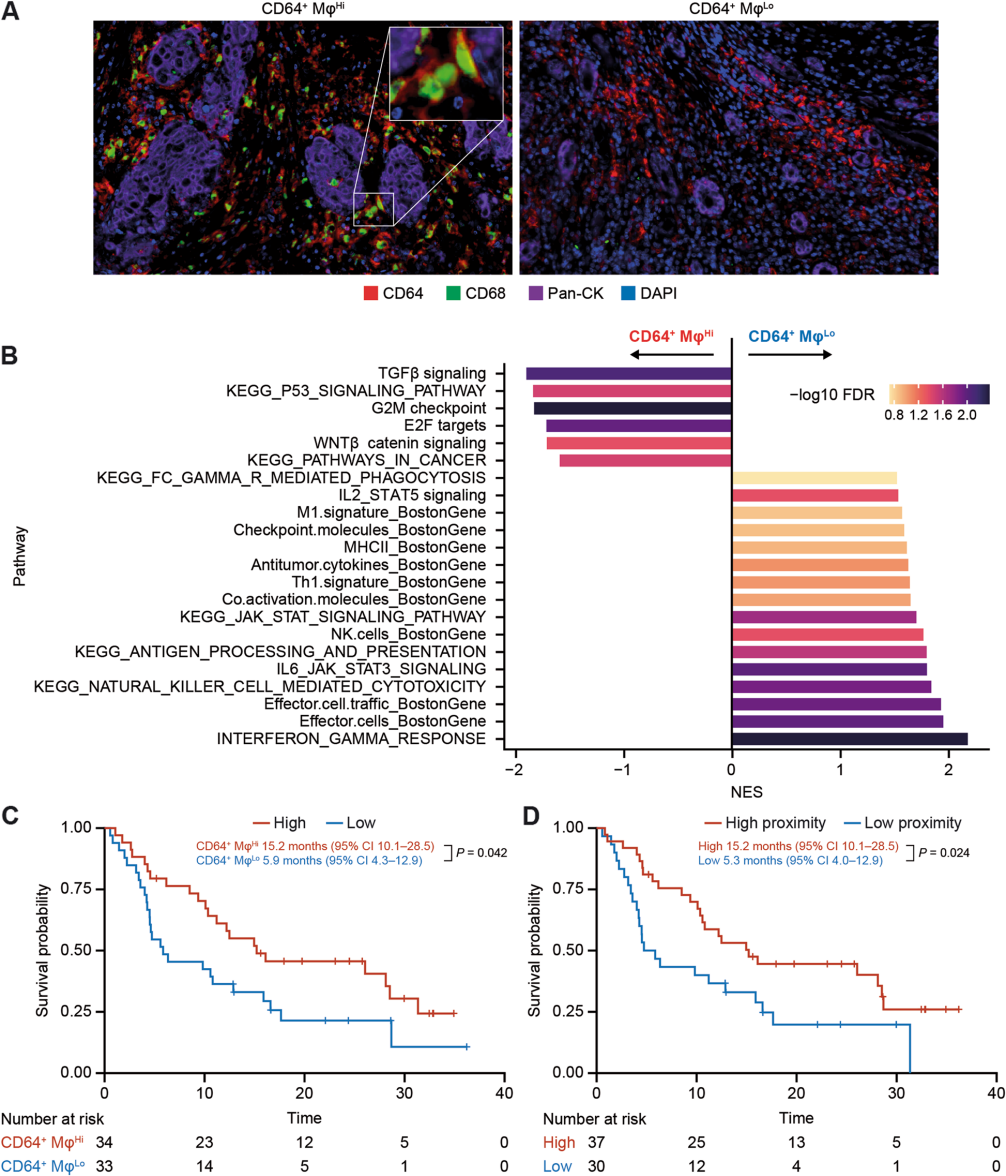
Association of CD64^+^ Mφ with immune-activated TME and clinical benefit of tislelizumab treatment in the mIHC BEP. **A** Representative mIHC image for a patient with a high and low CD64^+^ Mφ density. **B** GSEA illustrating the association of CD64^+^ Mφ density and TME pathways. **C** Kaplan–Meier overall survival analysis in subgroups defined by density of CD64^+^ Mφ. **D** Kaplan–Meier overall survival analysis in subgroups defined by proximity of CD8^+^ T cells and CD64^+^ Mφ. Median overall survival was estimated by the Kaplan–Meier method and the log-rank test was used to compare survival curves between defined biomarker subgroups. BEP, biomarker-evaluable population; CI, confidence interval; DAPI, 4’,6-diamidino-2-phenylindole; FDR, false discovery rate; GSEA, gene set enrichment analysis; Mφ, macrophages; mIHC, multiplex immunohistochemistry; NES, normalized enrichment score; Pan-CK, pan-cytokeratin; TME, tumor microenvironment

As co-enrichment with CD8^+^ T cells was associated with pro-inflammatory polarization of Mφ, it was hypothesized that pro-inflammatory Mφ may be associated with the OS benefit of tislelizumab. CD64, a surface marker, is typically induced on Mφ by inflamed signals, but the role of CD64^+^ Mφ in the TME has not been clearly defined. Using the latest published single-cell datasets [17], we analyzed the properties of CD64 expression within Mφ subgroups and found that CD64 was more likely to be expressed on pro-inflammatory C1QC^+^ or ISG15^+^ Mφ than on pro-angiogenic SPP1^+^ Mφ (Supplemental Fig. 3). To further confirm the role of CD64^+^ Mφ, GSEA was conducted to explore the association of CD64^+^ Mφ with TME pathways. The results revealed a strong correlation between CD64^+^ Mφ (CD64^+^CD68^+^) density and immune-activated pathways (e.g., IFNγ response, cytotoxic cells, antigen presentation signals) and a negative association with pro-tumor signals (e.g., TGFβ, Wnt, tumor proliferation signals) (Fig. 4A, B). Patients with a high density of CD64^+^ Mφ exhibited a longer median OS than those with a low density of CD64^+^ Mφ (15.2 vs. 5.9 months; *P* = 0.042; Fig. 4C). In addition, spatial proximity analysis revealed that patients with relatively high (closer) proximity between CD8^+^ T cells and CD64^+^ Mφ achieved longer median OS compared with those with low (further) proximity (15.2 vs. 5.3 months; *P* = 0.024; Fig. 4D). Consistently, patients with high proximity exhibited enrichment of multiple immune-activated pathways and higher expression of the T-cell trafficking chemokines CXCL9, CXCL10, CCL4, and CCL5 (Supplemental Fig. 4A, B).

Taken together, high CD64^+^ pro-inflammatory Mφ density was associated with an immune-activated TME and prolonged OS in patients who received tislelizumab.

## Discussion

Although CD8^+^ T cells alone have been reported as a potential predictive biomarker for immune checkpoint inhibitors, indicative of an immunologically “hot” tumor, the TME is an extremely complex system involving other key players. Therefore, a combination of biomarkers may have the capability to deliver multi-dimensional information and to provide additional insight into clinical use [33–35]. In the current study, we focused on Mφ in the TME, a long-standing major component with multiple controversial observations. For the first time, we found that co-enrichment of CD8^+^ T cells and Mφ was associated with OS benefit by both mIHC and GEP. Analysis of the TCGA pan-solid tumor dataset suggested our observation may be tislelizumab treatment-related rather than a prognostic factor.

By focusing on the functional gene expression profile of tislelizumab-treated patients, we found that the subgroup with high expression of both CD8^+^ T-cell and Mφ signatures exhibited a more immune-activating phenotype compared with subgroups with high CD8^+^ T-cell signature or high Mφ signature expression only, prompting us to speculate on the potential positive feedback loop between them. Firstly, pro-inflammatory Mφ-derived chemokines have been reported to attract circulating T cells to the TME [36, 37]. This is consistent with our finding that the subgroup with high CD8^+^ T-cell and Mφ signature expression exhibited increased expression of genes involved in T-cell recruitment, including *CXCL9*, the most significant gene associated with immunotherapy efficacy in a meta-analysis [38]. As one of the major antigen-presenting cell types in the TME, Mφ also directly activate helper T cells, which further promote the anti-tumor activity of cytotoxic T cells, as demonstrated in the present study by the enhanced expression level of the granzyme gene family. Together with the increased expression of the MHC I antigen presentation signature observed in our analysis, co-existence with pro-inflammatory Mφ created a TME conducive to increased CD8^+^ T-cell activation.

Reciprocally, CD8^+^ T cells may also drive pro-inflammatory polarization of monocytes/Mφ, as suggested by the higher expression of IFNγ signaling pathway-related genes such as *STAT1*, *ISG15,* and *IRF1* in patients with high CD8^+^ T-cell and Mφ signature expression in our analyses. In contrast, pro-angiogenic or M2-type-related genes such as *SPP1* and *TGFB2* were found to be expressed at higher levels by Mφ in the subgroup with low CD8^+^ T-cell signature expression and high Mφ signature expression. Therefore, high levels of both CD8^+^ T cells and Mφ may indicate the existence of a positive feedback loop that establishes a favorable baseline TME for immunotherapy. Indeed, this has been reported in an animal model and in patients with triple-negative breast cancer [39, 40]. High CD64-expressing pro-inflammatory Mφ (CD64^+^CD68^+^) density was also associated with OS benefit. Additionally, proximity between CD8^+^ T cells and pro-inflammatory CD64^+^ Mφ was associated with OS benefit in our analysis, suggesting an association with a CD8 co-enriched TME and further strengthening our crosstalk hypothesis and dual-biomarker strategy.

Although CD64 expression is correlated with pro-inflammatory Mφ, it may also be detrimental to effector T cells through antibody-FcγR binding-mediated ADCP. Previously, an inverse correlation between CD64^+^ Mφ infiltration and the density of CD8^+^ PD-1^+^ T cells was observed within tumors after treatment with Fc function-competent anti-PD-1 antibodies [23]. With its uniquely engineered Fc region, tislelizumab has the potential to avoid this adverse effect of effector T-cell depletion by CD64^+^ Mφ, while retaining their pro-inflammatory anti-tumor activity. As expected, we observed longer OS in tislelizumab-treated patients with a higher density of CD64^+^ Mφ and found that CD64 + Mφ density was positively associated with enrichment of immune-activating pathways (e.g., IFNγ response, cytotoxic cell, and antigen presentation), and negative association with pro-tumor signals (e.g., TGFβ, Wnt signaling, and tumor proliferation). Tislelizumab has previously demonstrated a high complete response rate regardless of FcγRΙ-expressing Mφ abundance in the classical Hodgkin lymphoma TME [41, 42], further supporting the lack of a negative effect of CD64^+^ Mφ infiltration on the anti-tumor activity of tislelizumab.

Further analysis of the biomarker strategy in this study is warranted and should be guided by the findings presented here. When assessing the results of this study, two aspects of the design should be considered: the small sample size of the mIHC dataset, which reduced statistical power for these analyses, and the absence of formal statistical hypothesis testing due to the retrospective nature of the analyses. The smaller sample size may have contributed to the non-significant OS result in the CD8^Hi^ subgroup vs. the CD8^Lo^ subgroup in the mIHC dataset, which was in contrast to results in the GEP dataset (CD8^hi^ vs. CD8^lo^, median OS 13.27 vs. 8.57 months, *P* = 0.0026) and prior studies [43]. The accuracy of the cell proportion estimations in the GEP dataset could have been improved by higher resolution bulk RNA-seq and, as a pan-cancer study. Additionally, for future studies, further exploration in each indication would be required because TME differences between tumor types were not considered in most analyses reported herein. Use of a multivariant model may have offered identification and analysis of confounding factors such as age, sex and prior therapies, and better evidence for our crosstalk hypothesis may have benefited from inclusion of dynamic TME changes after treatment, but these had to be omitted because post-treatment sampling was not feasible.

In conclusion, the combination of high CD8^+^ T-cell and high Mφ levels may aid the identification of the subset of patients who are most likely to benefit from treatment with tislelizumab. This strategy may provide new insights into the design of therapeutic antibodies and drug development in the future.

## Supplementary Information

**Additional file 1:** **Supplementary Figure 1.** Association of TME characteristics with survival benefit of tislelizumab treatment in the mIHC BEP. **Supplementary Figure 2.** Distinct TME in four mIHC-defined subgroups in the mIHC BEP. **Supplementary Figure 3.** CD64 expression in Mφ subtypes per single-cell sequencing data. **Supplementary Figure 4.** Proximity between CD64^+^ Mφ and CD8^+^ T cells and T-cell trafficking chemokine expression.

**Additional file 2.** Ethics committees and study approval numbers.

## Data Availability

On request, and subject to certain criteria, conditions, and exceptions, BeiGene, Ltd., will provide access to individual de-identified participant data from BeiGene-sponsored global interventional clinical studies conducted for medicines (1) for indications that have been approved or (2) in programs that have been terminated. BeiGene will also consider requests for the protocol, data dictionary, and statistical analysis plan. Data requests may be submitted to DataDisclosure@beigene.com.

## Abbreviations

ADCP: Antibody-dependent cellular phagocytosis
BEP: Biomarker-evaluable population
CD64^+^: CD64-positive
CD68^Hi^: High Mφ density
CD68^Lo^: Low Mφ density
CD8^+^: CD8-positive
CD8^Hi^: High CD8^+^ T-cell density
CD8^Lo^: Low CD8^+^ T-cell density
CI: Confidence interval
DAPI: 4’,6-Diamidino-2-phenylindole
EC: Esophageal cancer
ECOG: Eastern Cooperative Oncology Group
FcγR: Fc gamma receptor
FDR: False discovery rate
FFPE: Formalin-fixed, paraffin-embedded
GC: Gastric cancer
GEP: Gene expression profiling
GSEA: Gene set enrichment analysis
HCC: Hepatocellular carcinoma
HR: Hazard ratio
IC: Immune cells
IFNγ: Interferon-gamma
IgG: Immunoglobulin G
Mφ: Macrophage
MHC I: Major histocompatibility complex class I
mIHC: Multiplex immunohistochemistry
NA: Not available
NES: Normalized enrichment score
NSCLC: Non-small cell lung cancer
OS: Overall survival
Pan-CK: Pan-cytokeratin
PD-1: Programmed cell death protein 1
PD-L1: Programmed death-ligand 1
PD-L1 +: PD-L1‒positive
RNA-seq: RNA-sequencing
TCGA: The Cancer Genome Atlas
TME: Tumor microenvironment
UC: Urothelial cancer
