# Supplementary material for: Co-enrichment of CD8-positive T cells and macrophages is associated with clinical benefit of tislelizumab in solid tumors
Source: Biomark Res. 2023 Mar 7;11:25. doi: 10.1186/s40364-023-00465-w (PMC9990338; doi:10.1186/s40364-023-00465-w)

# Supplementary Material

Supplementary Figure 1. Association of TME characteristics with survival benefit of tislelizumab treatment **in the mIHC BEP**

A: Representative composite mIHC image showing the distribution and co-expression of individual markers (CD8, CD64, CD68, PD-L1, and Pan-CK) within a tumor region; A1: PD-L1+ tumor cell; A2: PD-L1+ Mφ; A3: CD64^+^ Mφ; A4: CD8^+^ T cell. Scale bar: 50 µm.

B–D: Kaplan-Meier overall survival analysis in tislelizumab-treated patients in subgroups defined by density of total PD-L1+ cells (B), PD-L1+ tumor cells (C), and PD-L1+ Mφ (D).

Median overall survival was estimated by the Kaplan-Meier method and the log-rank test was used to compare survival curves between defined biomarker subgroups.


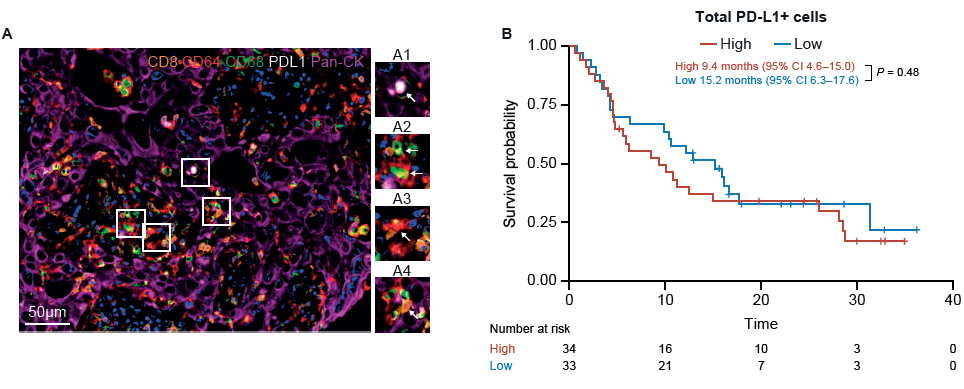
BEP, biomarker-evaluable population; CI, confidence interval; Mφ, macrophages; mIHC, multiplex immunohistochemistry; Pan-CK, pan-cytokeratin; PD-L1, programmed death-ligand 1; TME, tumor microenvironment.


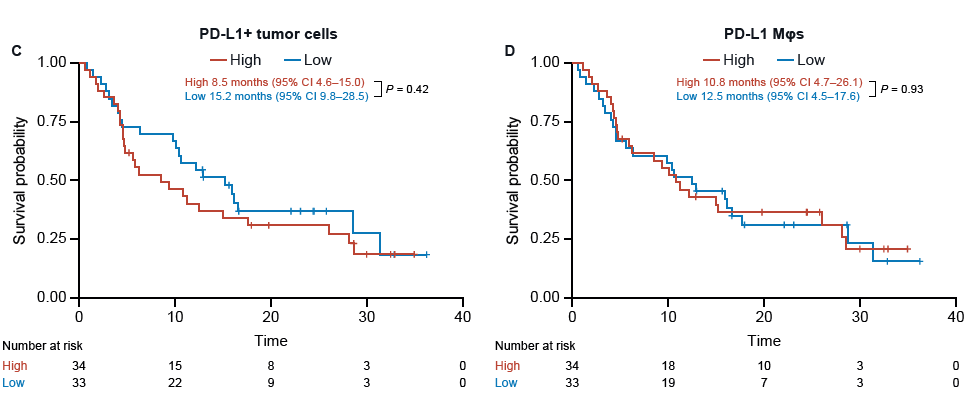


**PD-L1+ Mφs**

### Supplemental Figure 2. Distinct TME in four mIHC-defined subgroups in the mIHC BEP

A: Box plot showing signature score differences in cytotoxic T-cell, T-cell trafficking, MHC I, and tumor proliferation signatures among four subgroups defined by CD8^+^ T cell and CD68^+^ Mφ density.

### Supplementary Figure 2. Distinct TME in four mIHC-defined subgroups in the mIHC BEP

A: Box plot showing signature score differences in cytotoxic T-cell, T-cell trafficking, MHC I, and tumor proliferation signatures among four subgroups defined by CD8^+^ T cell and CD68^+^ Mφ density.

B: GSEA of pro-inflammatory Mφ polarization signals between CD8^Hi^/ Mφ ^Hi^ and CD8^Lo^/ Mφ ^Hi^ subgroups.
**P* < .05, ***P* < .01, ****P* < .001, *****P* < .0001.

BEP, biomarker-evaluable population; FDR, false discovery rate; GSEA, gene set enrichment analysis; Mφ, macrophages; mIHC, multiplex immunohistochemistry; MHC I, major histocompatibility complex class I; TME, tumor microenvironment.


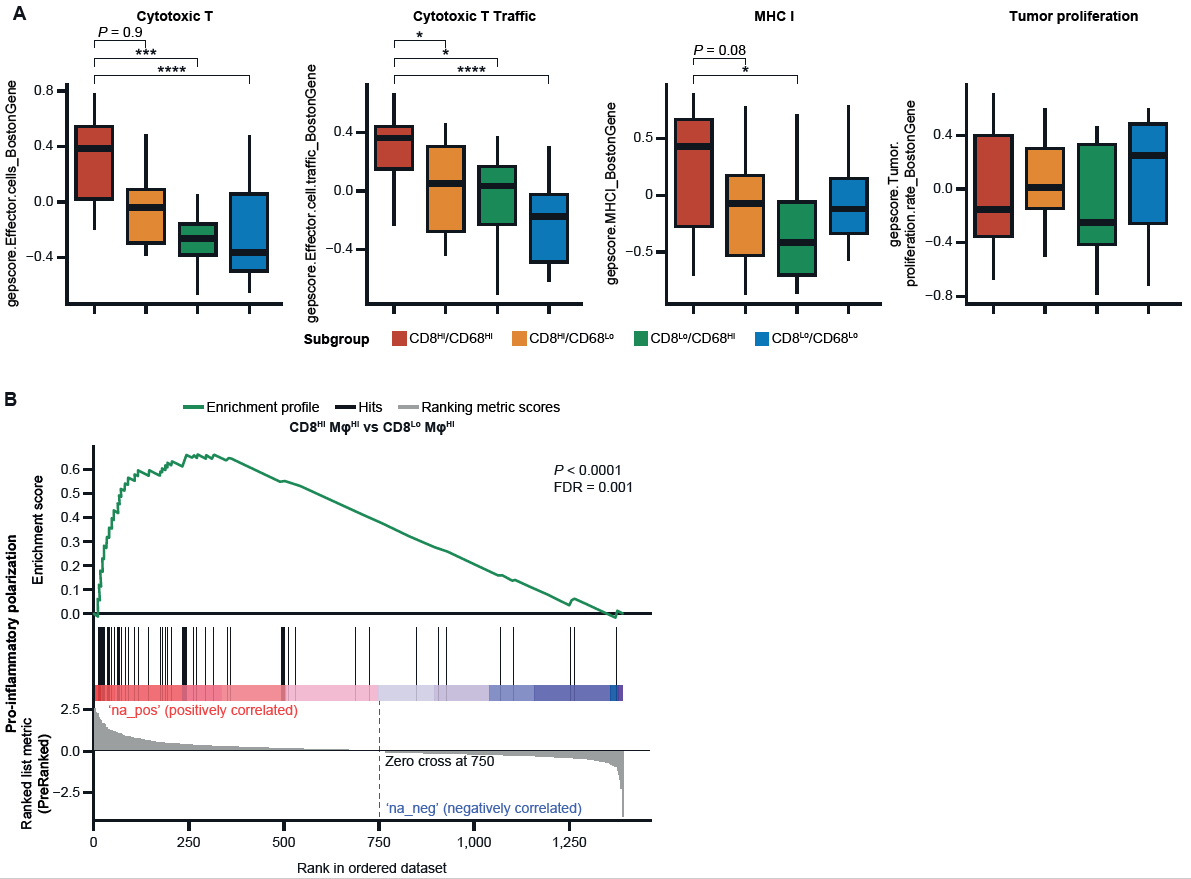


**Supplementary Figure** **3. CD64 expression in Mφ subtypes per single-cell sequencing data**

CD64 (FCGR1A) is enriched in pro-inflammatory Mφ rather than in pro-angiogenic Mφ across multiple solid tumors.
*Pro-angiogenic Mφ per the definition in the source data [17].


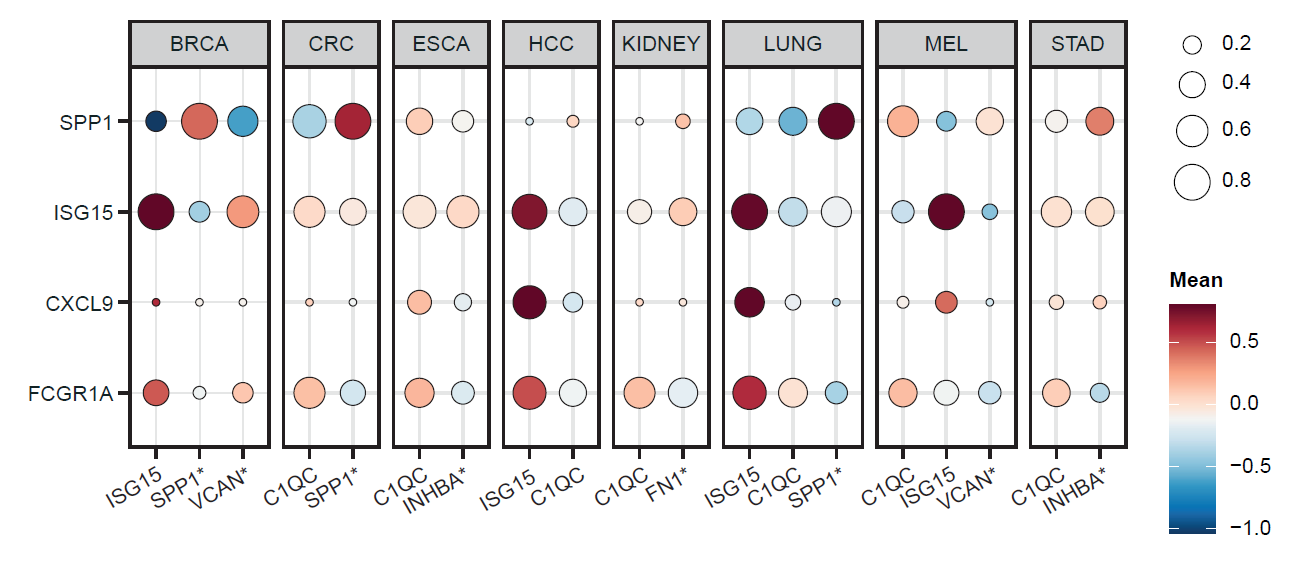
BRCA, breast cancer; CRC, colorectal cancer; ESCA, esophageal carcinoma; HCC, hepatocellular carcinoma; Mφ, macrophages; MEL, melanoma; STAD, stomach adenocarcinoma.

**Supplementary Figure 4. Proximity between CD64^+^ Mφ and CD8^+^ T cells and T-cell trafficking chemokine expression**

A: GSEA illustrating the TME signatures associated with high or low proximity between CD64^+^ Mφ and CD8^+^ T cells.

B: T-cell trafficking chemokine expression enriched in tumors with high proximity between CD64+ Mφ and CD8+ T cells.

GSEA, gene set enrichment analysis; Mφ, macrophages; NES, normalized enrichment score; TME, tumor microenvironment.


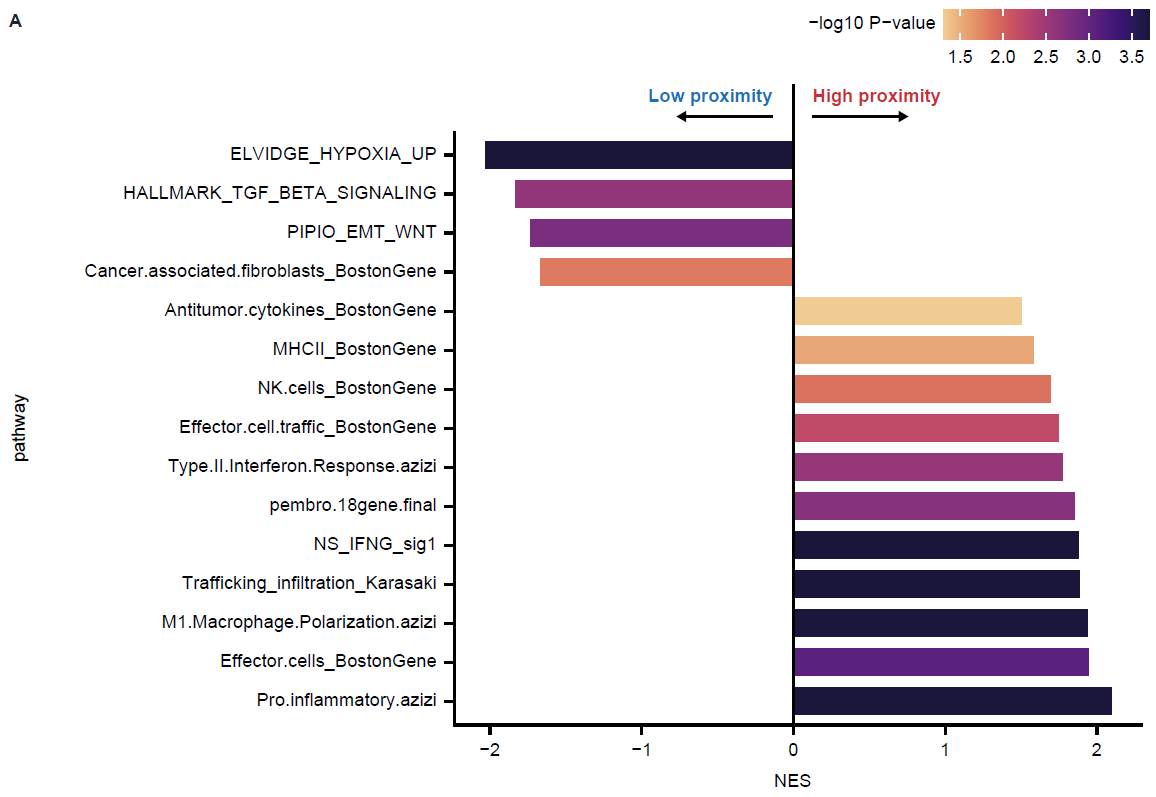

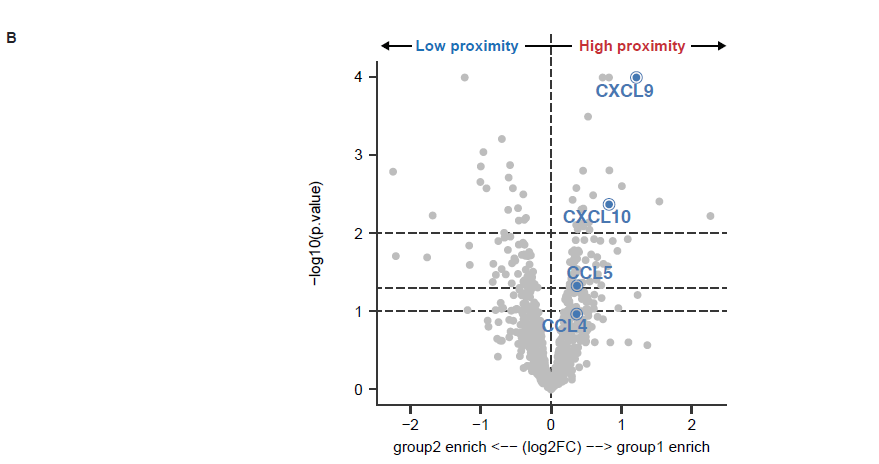

Supplement: Supplementary file 1 — Additional file 1: Supplementary Figure 1. Association of TME characteristics with survival benefit of tislelizumab treatment in the mIHC BEP. Supplementary Figure 2. Distinct TME in four mIHC-defined subgroups in the mIHC BEP. Supplementary Figure 3. CD64 expression in Mφ subtypes per single-cell sequencing data. Supplementary Figure 4. Proximity between CD64+ Mφ and CD8+ T cells and T-cell trafficking chemokine expression. [file 40364_2023_465_MOESM1_ESM.docx]
