# Supplementary material for: Co-enrichment of CD8-positive T cells and macrophages is associated with clinical benefit of tislelizumab in solid tumors
Source: Biomark Res. 2023 Mar 7;11:25. doi: 10.1186/s40364-023-00465-w (PMC9990338; doi:10.1186/s40364-023-00465-w)
Supplement: Supplementary file 2 — Additional file 2. Ethics committees and study approval numbers. [file 40364_2023_465_MOESM2_ESM.docx]

**Ethics committees and study approval numbers**

| **Study** | **Name of Ethics committee** | **Reference number** |
| --- | --- | --- |
| BGB-A317-001 | Royal Melbourne Hospital | HREC/15/MH/77 |
| BGB-A317-001 | Linear Clinical Research Limited | 2015-03-206-AA |
| BGB-A317-001 | Peter MacCallum Cancer Center | HREC/15/MH/77 |
| BGB-A317-001 | The Austin Hospital | HREC/15/MH/77 |
| BGB-A317-001 | Monash Medical Centre-Clayton Campus | HREC/15/MH/77 |
| BGB-A317-001 | Prince Wales Hospital | HREC/15/MH/77 |
| BGB-A317-001 | Nucleus Network | 490/15 |
| BGB-A317-001 | Tasman Oncology Research Ltd | 2015-03-206-AA |
| BGB-A317-001 | Royal Adelaide Hospital | HREC/15/TQEH/194 |
| BGB-A317-001 | Chris O'Brien Lifehouse | HREC/15/CRGH/232 |
| BGB-A317-001 | Princess Alexandra Hospital | HREC/15/MH/77 |
| BGB-A317-001 | Cabrini Hospital Malvern | 04-18-01-16 |
| BGB-A317-001 | The Queen Elizabeth Hospital | HREC/15/TQEH/194 |
| BGB-A317-001 | Wellington Hospital | 15/CEN/179 |
| BGB-A317-001 | Auckland City Hospital | 15/CEN/179 |
| BGB-A317-001 | Waikato Hospital | 15/CEN/179 |
| BGB-A317-001 | Massachusetts General Hospital | 16-183 |
| BGB-A317-001 | Oncology Consultants, P.A | 1173356 |
| BGB-A317-001 | Seoul National University Hospital | H-1603-162-753 |
| BGB-A317-001 | Asan Medical Center | S2016-0492-0001 |
| BGB-A317-001 | Seoul National University Bundang Hospital | B-1605/345-001 |
| BGB-A317-001 | National Taiwan University Hospital | 201603027MSD |
| BGB-A317-001 | National Cheng Kung University Hospital | AB-CR-105-023 |
| BGB-A317-001 | Chang Gung Memorial Hospital, Chiayi | 201600894A4 |
| BGB-A317-001 | Kaohsiung Chang Gung Memorial Hospital | 201600894A4 |
| BGB-A317-001 | Taipei Veterans General Hospital | 2016-09-001CU |
| BGB-A317-001 | Chang Gung Memorial Hospital, Linkou | 201600894A4 |
| BGB-A317-204 | Ethics Committee of Fudan University Shanghai Cancer Center, No. 270, Dongan Road, Xu Hui District, Shanghai, 200032, P.R.China | 伦理意见通知函 20170227 1702169-8-1703 1702169-8-1709A 1702169-8-1805B 1702169-8-1806C 1702169-8-1808D 1702169-8-1811E |
| BGB-A317-204 | Ethics Committee of Cancer Hospital Chinese Academy of Medical Sciences, 17 Panjiayuan South, Chaoyang District, Beijing, China, 100021 | 审批号17-005/1259 (2017-3-6) 审批号17-005/1259 (2017-4-12) 2017 年8 月25 日 2018 年4 月13 日 2018 年6 月8 日 2018 年9 月14 日 |
| BGB-A317-204 | Ethics Committee of Peking Union Medical College Hospital, No. 41, Damucang Hutong, Xicheng District, Beijing, China | HS2017098 KS2018016 KS2018128 KS2018192 KS2018491 |
| BGB-A317-204 | Biomedical Research Ethics Committee of Peking Unversity First Hospital, No.6, Da Hong Luo Chang Street, Xicheng District, Beijing, China | （2017）药物注册第（25）号/2017-04-19 （2017）药物注册第（25）号/2017-05-17 （2017）药物注册第（25）号/2017-06-14 （2017）药物注册第（25）号-修正案/2017-08-30 （2017）药物注册第（25）号-修正案/2018-02-07 （2017）药物注册第（25）号-修正案/2018-06-27 （2017）药物注册第（25）号-修正案/2018-08-01 （2017）药物注册第（25）号-修正案/2018-09-12 （2017）药物注册第（25）号-修正案/2019-01-02 |
| BGB-A317-204 | Ethics Committee of Medical Science Research in Peking University Third Hospital, No.49 Garden North Road, Haidian District, Beijing, China, 100083 | （2017）药伦审第（010-01）号/20170209 （2017）药伦审第（010-02）号/20170406 （2017）药伦审第（010-03）号/20170815 （2017）药伦审第（010-04）号/20170925 （2017）药伦审第（010-05）号/20180413 （2017）药伦审第（010-06）号/20180614 （2017）药伦审第（010-07）号/20181226 |
| BGB-A317-204 | Ethics Committee of Zhongshan Hospital affiliated to Fudan University, No. 180, Fenglin Road, Xuhui District, Shanghai, China， 200032 | 伦理委员会通知函20170410 2017-018R/20170514 2017-018（2）/20171025 2017-018（3）/20180709 2017-018（4）/20181015 |
| BGB-A317-204 | Ethics Committee of Jiangsu Cancer Hospital, NO 42 baiziting， xuanwumen，najing City, Jiangsu Province, China, 210009 | 2017-004/20170301 2017-004-02/20170629 2017-004-03/20170929 2017-004-04/20180731 2017-004-05/20180926 |
| BGB-A317-204 | Ethics Committee of Zhejiang Cancer Hospital, No.1, Banshan Dong Lu, Gongshu District, Hangzhou City, Zhejiang Province, China | IRB-[2017]6 号 IRB-[2017]91 号 IRB-[2017]214 号 IRB-[2018]94 号 IRB-[2018]188 号 IRB-[2018]442 号 |
| BGB-A317-204 | Ethics Committee of The First Affiliated Hospital Of Nanchang University, No.17 Yongwaizhen Street,Nanchang,Jiangxi,China | 【2017】临伦审第007 号 【2017】临伦审第（007）-1 号 【2017】临伦审第（007）-2 号 【2017】临伦审第（007）-3 号 【2017】临伦审第（007）-4 号 |
| BGB-A317-204 | Ethics Committee of Sun Yat-sen Memorial Hospital, Sun Yat-sen University, No. 107, Yanjiang West Road, Yuexiu District, Guangzhou City, Guangdong Province, China 510120 | [2017]伦审药第（20）号 2017 快审第（43）号 [2018]伦审药第（48）号 2018 快审第（55）号 2018 快审第（142）号 2018 快审第（172）号 |
| BGB-A317-204 | Clinical Trial Ethics Committee of West China Hospital of Sichuan University, No. 37, Guoxue Road, Wuhou District, Chengdu City, Sichuan Province, China 610041 | 2017 年临床试验（西药）审（29）号/2017-03-22 2017 年临床试验（西药）审（29）号/2017-05-17 伦理备案批准函2017-10-18 伦理意见通知函2018-05-07 伦理备案批准函2018-09-03 伦理备案批准函2019-01-04 |
| BGB-A317-204 | Clinical Trial Ethics Committee of Huazhong University of Science and Technology, No.13 Hangkong Road, Wuhan, China | 伦理意见通知函20170329 【2017】伦审字（45）号 【2017】伦审字（45）-1 号 【2017】伦审字（45）-2 号 【2017】伦审字（45）-3 号 【2017】伦审字（45）-4 号 |
| BGB-A317-204 | Ethics Committee of Fujian Medical University Union Hospital, No.29 Xinquan Road, Gulou District, Fuzhou City, Fujian Province, China 350001 | 2017YW020-01 2017YW020-02 2017YW020-03 2017YW020-04 2017YW020-05 2017YW020-07 |
| BGB-A317-204 | Clinical Trial Ethics Committee of The first affiliated of Wenzhou medical university, Nanbaixiang, ouhai district,Wenzhou City, Zhejiang Province, China 325000 | 伦理意见通知函YJ2017-016-01 临床研究（药/械）伦审（2017）第（029）号 临床研究（药/械）伦审（2017）第（088）号 临床研究（药/械）伦审（2018）第（048）号 临床研究（药/械）伦审（2018）第（082）号 临床研究（药/械）伦审（2018）第（101）号 |
| BGB-A317-204 | Ethics Committee of The Second Affiliated Hospital of Xi’an Jiaotong University, No.30 ,Huangcheng West Road, Xi 'an City, China | （2017）伦审第（011）号/20170519 2017-09-08 2018-04-19 2018-07-26 2018-07-26 2018-09-13 |
| BGB-A317-204 | Ethics Committee of Hunan Cancer Hospital, No. 283, Tongzipo Road, Yuelu District, Changsha City, Hunan Province, China, 410013 | 2017 年药审[93]号 2017 年快审[117]号 2018 年快审[96]号 2018 年快审[160]号 2018 年药审[179]号 |
| BGB-A317-204 | Ethics Committee of The first hospital of China medical university, No.155, Nanjing North Street, Heping District, Shenyang, China, 110001 | 会议审查修改意见反馈20170627 2017YL031 2017YL031-1 2017YL031-2 2017YL031-3 2017YL031-4 2017YL031-5 |
| BGB-A317-204 | Ethics Committee of Anhui Provincial Hospital, No. 17, lujiang road, Luyang District, hefei, Anhui Province, China, 230001 | 伦理意见通知函20170710 2017 伦审第142 号 2017 伦审第147 号 2017 伦审第187 号 2017 伦审第57 号 2017 伦审第132 号 2017 伦审第223 号 |
| BGB-A317-204 | Ethics Committee of Jiang Xi Cancer Hospital, No. 519, Beijing east road, Nanchang, Jiangxi Province, China, 330029 | 伦理意见通知函20170509 2017012-YW004/2017-6-23 2017012-YW004/2017-7-14 2017012-YW004/2017-8-22 伦理意见通知函20180521 2017012-YW004/2018-8-6 |
| BGB-A317-204 | Ethics Committee of Samsung Medical Center, 81, Irwon-ro, Gangnam-gu, Seoul, 06351, South Korea | N/A |
| BGB-A317-204 | Ethics Committee of Seoul National University Hospital, 101, Daehak-ro, Jongno-gu, Seoul, 03080 | N/A |
| BGB-A317-204 | Ethics Committee of Severance Hospital, Yonsei University Health System, 50-1 Yonsei-ro, Seodaemungu, Seoul, 03722 | N/A |
| BGB-A317-204 | Ethics Committee of Liaoning Cancer Hospital, No. 44, Xiaoheyan Road, Dadong District, Shenyang, P. R, China, 110042 | 20170504 20180134 201803731 20180386 20180614 20180611 20180722 20181006 |
| BGB-A317-204 | Ethics Committee of The Second Hospital of Tianjin Medical University, No. 23 Pingjiangdao, Hexi District, Tianjin, China, 300211 | 临审【2017】第（012）号 2017K033 2018K017 2018K021 2018K024 2018K051 |
| BGB-A317-204 | Ethics Committee of The Second Hospital of Dalian Medical University, No. 467, Zhongshan Road, Shahekou District, Dalian, P. R, China, 116023 | 大医二院伦审2017 第111 号 大医二院伦审2019 第011 号 |
| BGB-A317-204 | Ethics Committee of Zhejiang Provincial People's Hospital, No.158 Shangtang Road,Hangzhou,Zhejiang Province,China,310000 | 2017YW017-1 10/30/2017-2 10/30/2017-3 4/23/2018-4 6/13/2018-5 7/30/2018-6 10/25/2018-7 |
| BGB-A317-204 | Clinical Trial Ethics Committee of the First Affiliated of Xiamen University, N0 55 zhenhai road, siming district, xiamen City, Fujian Province, China 361003 | XMYY-2017Y025-01 （2017）药伦审字（030）号 XMYY-2017Y025-04 XMYY-2017Y025-05 XMYY-2017Y025-06 XMYY-2017Y025-07 XMYY-2017Y025-08 XMYY-2017Y025-09 XMYY-2017Y025-10 |
| BGB-A317-204 | Ethics Committee of East China Hospital Affiliated to Fudan University, No.168, Yan 'an West Road, Shanghai, China | 伦理意见通知函2017L011 AF16 伦理批件（快速审查）2017L011 AF16 伦理批件（快速审查）2017L011-X181 AF16 伦理批件（快速审查）2017L011-X182 AF16 伦理批件（快速审查）2017L011-X183 |
| BGB-A317-204 | Ethics Committee of Xiangya Hospital, Central South University, No. 87, Xiangya Road, Kaifu District, Changsha City, Hunan Province, China 410008 | 伦审快第（201707082）号 伦审快第（201708089）号 伦审快第（201806080）号 伦审第（201707091-2）号/2018-7-30 伦审第（201707091-2）号/2018-10-18 |
| BGB-A317-204 | Ethics Committee of The First Affiliated Hospital Of Dalian Medical University, No.222 Zhongshan Road, Dalian, Liaoning Province, China, 116011 | 伦理意见通知函PJ-JG-2017-103 PJ-JG-2017-103(X) YJ-JG-YW-2018-78 |
| BGB-A317-102 | Gunagdong General Hospital | 2016 55 |
| BGB-A317-102 | Beijing Cancer Hospital | 2016YW62 |
| BGB-A317-102 | Beijing Cancer Hospital | 2016YW63 |
| BGB-A317-102 | The Harbin Medical University Cancer Hospital | 2016-61 |
| BGB-A317-102 | The Harbin Medical University Cancer Hospital | 2016-62 |
| BGB-A317-102 | Zhongshan Hospital Fudan University | 2017-002（3） |
| BGB-A317-102 | The 2nd affiliated Hospital School of Medicine, Zhejiang University | 2017伦审药第（252）号 |
| BGB-A317-102 | Beijing Cancer Hospital | 2016YW72 |
| BGB-A317-102 | Cancer hospital Chinese academy of medical science | N/A |
| BGB-A317-102 | Jiangsu Province People’s Hospital | 2016-MD-227.A1 |
| BGB-A317-102 | Sir Run Run Shaw Hospital School of Medicine, Zhejiang University | 20170920-2 |
| BGB-A317-102 | Fudan University Shanghai Cancer Center | 1612167-2-1804A |
| BGB-A317-102 | Cancer hospital Chinese academy of medical science | N/A |
| BGB-A317-102 | The Fifth Affiliated Hospital, Sun Yat-sen University | 中大五院【2017】伦字第（Y56-1）号 |
| BGB-A317-102 | Sun Yat-Sen Memorial Hospital, Sun Yat-Sen University | 2017快审第（61）号 |
| BGB-A317-102 | The First Affiliated Hospital of Nanchang University | 【2017】临伦审第071号 |
